# The Nomadic Bug: A Case Report of Salmonella Septic Arthritis of Sternoclavicular Joint in a Healthy Patient

**DOI:** 10.7759/cureus.57685

**Published:** 2024-04-05

**Authors:** Siti Nor Suzeana Mustfar, Raihanah Haroon, Azian Abd Aziz

**Affiliations:** 1 Radiology, Kulliyyah of Medicine, International Islamic University Malaysia, Kuantan, MYS

**Keywords:** healthy patient, ct-guided percutaneous drainage, salmonella septic arthritis, sternoclavicular septic arthritis, septic arthritis

## Abstract

In an otherwise healthy adult, septic arthritis of the sternoclavicular joint is very uncommon. Usually, individuals with a history of intravenous drug usage or those with impaired immune systems are affected. The usual mode of spread is hematogenous spread or direct spread via neighbouring sources of infection.

We report a rare case of mediastinitis and lung empyema preceded by sternoclavicular septic arthritis in an otherwise healthy 49-year-old woman due to Salmonella sp. Radiological imaging showed left sternoclavicular joint collection with bone destruction. The literature only contained reports of two prior occurrences of sternoclavicular joint septic arthritis caused by Salmonella. If diagnosed early, patients usually respond to medical treatment such as aspiration and antibiotics, as was the case with our patient.

## Introduction

In an adult who is otherwise healthy, septic arthritis of the sternoclavicular joint (SCJ) is very uncommon. Less than 0.5% of all bone and joint infections are said to affect this specific region [1, 2]. Patients with a history of intravenous drug misuse [3] or those with impaired immune systems [4] are typically affected. However, in the minority of patients, no risk factors were present. Patients with septic arthritis of the SCJ usually present with fever, pain, and local swelling. To avoid morbidity and mortality, sternoclavicular joint septic arthritis must be treated right away. Osteomyelitis, chest wall abscess, and mediastinitis are some of the condition's serious complications [5]. Depending on the severity and extent of the disease, the current treatment of choice is intravenous antibiotics, incision and drainage, surgical debridement or en-bloc resection.

This article was previously presented as a meeting poster at the 2021 Virtual Medical Research Symposium on December 14, 2021.

## Case presentation

A 49-year-old woman who never had any medical conditions before, came to our medical centre with a two-week history of abruptly developing left shoulder pain that radiated to the left neck and upper chest. She had a fever for a single day. There was no past medical history of trauma or neck interventions. Any movement of the left upper limb exacerbated the agony. She otherwise did not experience dyspnea. There were no noticeable skin changes or swelling palpable in the area of the left chest wall. She could not sleep at all because of the ache. She also experienced fullness in the left upper sternal area, but visible swelling was apparent.

**Figure 1:**
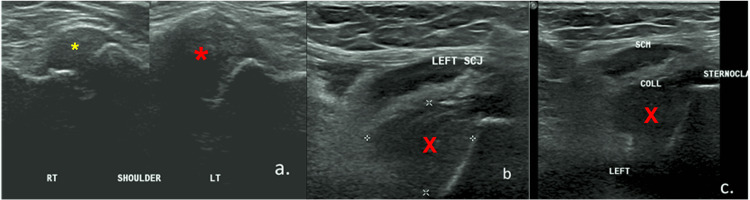
(a) Ultrasound of the sternoclavicular joint showed a distended capsule on the left side (big red asterisk) when compared to the right (small yellow asterisk). (b & c) Ultrasound revealed a collection (red 'X') inferior to the left sternoclavicular joint which extended into the left thoracic region.

Upon examination, the left sternal notch region showed some tenderness. No cervical lymph node could be felt. An X-ray of the cervical spine showed no abnormalities. The initial chest X-ray revealed clean lung fields throughout, with otherwise left upper zone opacities. A collection inferior to the left SCJ that extended into the left thoracic region with capsular distension was found during an ultrasound neck examination (Figure 1a-1c).

**Figure 2:**
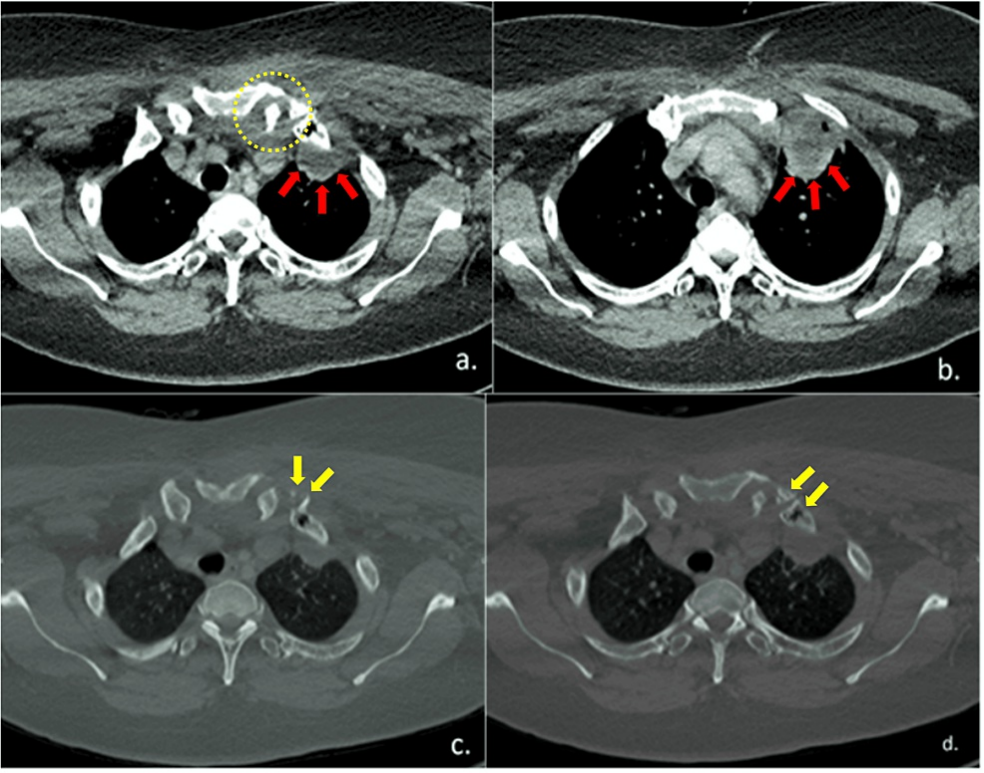
(a & b) CECT thorax showed a collection (yellow dotted circle) inferior to the left sternoclavicular joint and posterior to the left anterior first rib associated with left apical pleural abscess (red block arrows). (c & d) CECT thorax in the bone window revealed multiple lytic areas at the sternal end of left first rib associated with probable intraosseous air pockets/vacuum phenomenon in the 1st rib at costosternal junction (yellow block arrows).

The collection inferior to the left SCJ joint observed on ultrasound was further verified by a contrast-enhanced computed tomography (CECT) of the neck and thorax. Additional radiological findings of a left apical pleural collection and mediastinitis were also discovered (Figure 2a-2b). Figure 2b-2c also showed many lytic areas at the sternal end of the left first rib, which indicated bone destruction. Correlating with the brief clinical history, the CT appearances were suggestive of an inflammatory or infective condition. A possible diagnosis of septic arthritis of the left sternoclavicular joint associated with left lung empyema was made.

**Figure 3:**
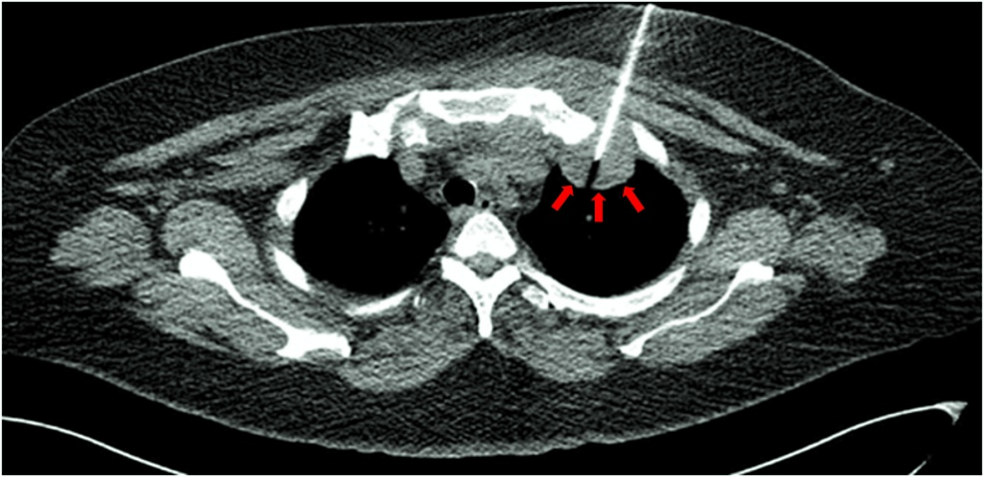
CT-guided aspiration of the left apical lung collection (red block arrows). The aspirate was sent for microscopy, culture and sensitivity and showed Salmonella group organisms.

As soon as the patient was admitted, intravenous amoxicillin clavulanate as an empirical antibiotic was administered. The pleural collection was aspirated the following day under CT guidance (Figure 3). After being aspirated, 8-10 millilitres of pus mixed with blood were sent for sensitivity testing, microscopy, and culture. The findings of the cytologic analysis showed organisms belonging to the Salmonella group without any indication of malignant cells. Bacteraemia from the Salmonella group was also detected in blood cultures. Tuberculous test results were negative. The patient's condition improved, and the patient was given antibiotics to go home with. In addition to scheduling a second CECT thorax for reevaluation, oral antibiotics were also administered for a duration of six weeks.

**Figure 4:**
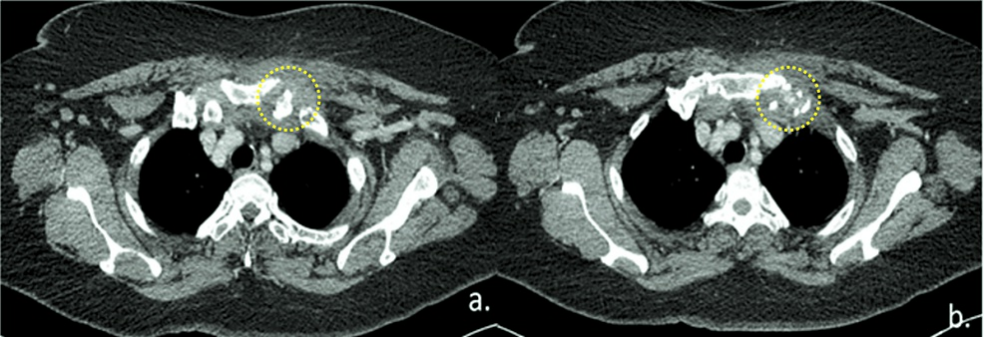
(a & b) Repeat CECT thorax seven weeks later revealed resolution of the left apical pleural collection with minimal residual left sternoclavicular joint collection and bone destruction (yellow dotted circles).

About seven weeks later, a follow-up CECT thorax (Figure 4) showed that the left apical pleural collection had resolved and that there was minimal residual left SCJ collection. During the subsequent follow-up visit, she stopped complaining of chest and shoulder pain. At the seven-week follow-up, C-reactive protein (CRP) and erythrocyte sedimentation rate (ESR) had also decreased. The patient had responded well to nine weeks of antibiotics, and after four weeks, the patient was scheduled for another follow-up.

## Discussion

SCJ is a rare location for septic arthritis owing to less than 0.5% in healthy individuals [1, 2]. Risk factors for SCJ septic arthritis include trauma, diabetes, intravenous drug use, infection at distant sites and infected central venous line [1, 2]. A previous case by Sharif et al. (2018) reported malignancy-related immunosuppression and late infection of the previous tracheostomy tract as possible culprits [6]. Whereas Womack (2012) reported two cases of urinary tract infection causing haematogenous spread of infection to SCJ [7]. However, in the minority of patients, about 23% presented with no risk factors. The usual causative pathogen is Staphylococcus aureus accounting for about 49%, followed by Pseudomonas aeruginosa (10%), Brucella melitensis (7%), and Escherichia coli (5%) [2]. The usual method of spreading is through haematogenous spread or from direct extension via adjacent sources of infection. In our patient, given the absence of any preceding risk factor, it is likely from haematogenous spread as she has a positive blood culture of the Salmonella group organism.

Non-typhoid Salmonella is the most common causative pathogen for gastrointestinal infection worldwide. Most patients with non-typhoid Salmonella infection have self-limiting symptoms which usually do not require antibiotics. Common symptoms are fever, abdominal discomfort, nausea and vomiting as well as diarrhoea. Salmonella bacteraemia occurs in about 5-10% of infected patients and some may develop focal infections such as meningitis, bone and joint infections. Immunocompromised patients may have a higher rate of complications and prolonged or recurrent salmonella infection [8]. Extra-intestinal salmonella infections may present as pneumonia, meningitis, mycotic aneurysm, osteomyelitis, septic arthritis, or cholangitis. This is especially true for patients who have chronic medical conditions including diabetes, hypertension, connective tissue disease, chronic lung disease, or cancer [9].

Antibiotics are typically not necessary for patients with Salmonella gastrointestinal infections due to the self-limiting course of the disease. The patient's general condition and the strain's susceptibility pattern determine which medications are best for them when they have an extra-intestinal localised infection. Third-generation cephalosporins, trimethoprim-sulfamethoxazole, ampicillin, and fluoroquinolones are among the options [9].

Individuals who have SCJ septic arthritis typically exhibit pain, localised swelling, and fever. Neck pain is an infrequent additional presentation for these patients, accounting for only 2% of the cases [2]. About 60% of the cases are unilateral, usually involving the right sternoclavicular joint [10]. From an anatomy perspective, several vital structures such as the subclavian vessels and the phrenic nerve lie in close proximity to the SCJ. Hence, infections affecting the SCJ structures should be treated urgently to avoid spreading and harming the important neighbouring structures [11].

The preferred methods for assessing the degree of SCJ septic arthritis and any local complications, as well as for directing the surgical approach, are computed tomography (CT) or magnetic resonance imaging (MRI) [2]. Mediastinitis, joint effusion, joint destruction, and additional complications like empyema or chest wall abscess will be readily detected using CT or MRI imaging. The aspirated pleural fluid culture and sensitivity confirmed the diagnosis of this patient, though this can also be achieved by open or tru-cut biopsy, aspirated joint fluid culture, or associated abscess [10]. In our patient, CT scan helped to confirm the ultrasound findings and to assess the extent of the collection and associated complications as well as to aid the aspiration of the pleural abscess. The significant joint or capsule distension extending to the sternum and clavicle was strongly suggestive of infection rather than a degenerative process [12].

The current therapy choices include intravenous antibiotics, incision and drainage, surgical debridement, or en-bloc resection, depending on the severity and extent of the disease. In the event of osteomyelitis and abscess, sternoclavicular joint excision and pectoralis flap closure are the usual surgical interventions [13]. Intravenous antibiotics may be all that is necessary for certain people, while more invasive measures may be required for others. As was the case with our patient, patients typically respond to medical care when discovered early, including aspiration and antibiotics.

## Conclusions

Salmonella septic arthritis of the SCJ is extremely rare in an otherwise healthy adult. Given the list of potential problems complicating this condition, early detection with prompt antibiotic administration is crucial to halt the disease progression and minimise further grave complications. Treating physicians should have a high index of suspicion of this condition and request the necessary radiological imagings to confirm the diagnosis. The associated lung empyema which complicated the condition further could be detrimental if not detected early and treated with appropriate surgical and antibiotics treatment.
